# Author’s reply

**DOI:** 10.4103/1817-1737.69120

**Published:** 2010

**Authors:** Salim Baharoon

**Affiliations:** *Intensive Care Departments, King Saud Bin Abdulaziz, University for Health Sciences, Saudi Arabia. E-mail: baharoon@hotmail.com*

Sir,

“Wiwanitkit highlighted few notes on the publication” H1N1 infection induced thyroid storm including weather this is the first reported case in litraure and the association between H1N1 infection and the thyroid storm.[1] Infection is frequently quoted as a possible precipitator to thyroid storm. This was highlighted both in the introduction and the discussion of our article. The mechanism by which systemic infections precipitate a storm is not well described in the literature.[2–8] We are not suggesting that the activation (induction of the storm) occurs because of direct invasion of the virus into the thyroid gland. The word induction surely is not meant to establish that case of correlation.

This case was published in the indexed *Annals of Thoracic Medicine* on April 14, 2010. Therefore, to our knowledge, we still believe that it is the first case to appear in the English literature that correlates H1N1 with thyroid storm. The case presented by Oguz *et al*. is quite interesting, however, it was presented in the abstract book of the “European Congress of Endocrinology” that was held between April 24 and 28, 2010, in Czech Republic, that is, after our publication date.
